# Intact face detection in young patients with major depressive disorder revealed by the face-specific N170 component

**DOI:** 10.1038/s41598-022-18491-3

**Published:** 2022-08-18

**Authors:** Yang Chen, Feizhou Tong, Lun Zhao, Gang Sun

**Affiliations:** 1 Department of Neurology, The 904th Hospital of PLA, Medical School of Anhui Medical University, Wuxi, China; 2 State Key Lab of Space Medicine Fundamentals and Application, China Astronaut Research and Training Center, Beijing, China; 3 Brain Science Center, Qingdao Tenocom Medical Technology Co. Ltd, Qingdao, China; 4 The Department of Medical Imaging, The 960th Hospital of Joint Logistics Support Force of PLA, Jinan, China

**Keywords:** Physiology, Psychology, Biomarkers

## Abstract

It is unclear whether the face perceptual processing, especially the perceptual computation in early stages of processing faces, impaired in young patients with major depressive disorder (MDD). In this study, the face perception was investigated by analyzing the early ERP components in response to upright and inverted faces versus objects. Across all conditions, both the P1 and the N170 components were similar in MDD patients versus in the controls, regardless of latencies or amplitudes. Faces elicited larger N170 than objects (N170 face effect) and inverted faces elicited higher and delayed N170 (N170 inversion effect); however, none of these effects were modulated by depression. These findings suggest that there is intact perceptual mechanism of processing faces in young MDD patients, relying primarily on global/configural information versus non-face objects.

## Introduction

The dysfunction of processing emotional information has been reported widely in patients with major depressive disorder (MDD), which may be the core symptom of disturbed mental state and interpersonal behavior and one of the reasons for the occurrence and maintenance of MDD. According to the cognitive theory of depression, in the process of the onset, maintenance and recurrence of MDD, there is cognitive preference in the process of emotional stimulation, which plays an important role in the development and persistence of depression^1–3^. For example, Surguladze et al.^4^ found that compared with the control group, MDD patients were less likely to label 50% of distorted happy faces with happiness, indicating that MDD patients had difficulty in recognizing mild happy expressions^4^. Using the forced choice task, Joormann and Gotlib^2^ showed that MDD patients could not judge subtle happy expressions as stronger than neutral and negative expressions and that when happy expressions and negative expressions (such as anger, fear or sadness) appeared at the same time, MDD patients were less likely to choose happy faces as stronger emotional expression, indicating that the difficulty of positive emotion detection in MDD patients may lead to their feeling lack of strength and reduce their approach behavior^2^.

As one of important ecological stimuli as well as the basis of interpersonal communication, faces can provide important clues relevant to human identity, emotion and perception information. Therefore, it has been investigated widely changes of face processes in MDD patients, with the finding of the emotional perception deficit reflected by the N170 abnormalities indexing non-specific facial affect processing as one of social impairments being broadly characteristic in neurological and psychiatric disorders^5,6^. For example, Dai and Feng found smaller N170 amplitudes to emotional faces in MDD patients^7^; in contrast, other investigations did not find amplitude or latency differences for the N170 between MDD patients and healthy controls^8–10^. Interestingly, although previous studies have indicated that in addition to deficits in recognizing positive and/or negative emotional faces, MDD patients also exhibited deficits in recognizing emotionally neutral faces^11–13^. For example, to test the hypothesis that MDD patients may attribute stimuli with different emotional valence to stimuli usually interpreted as neutrally emotional faces, Leppanen et al.^11^ asked participants to make forced-selection response to the transient neutral, happy and sad faces and found that the recognition accuracy of neutral faces in the control group was the same as that of happy and sad faces, but the recognition accuracy of neutral faces in MDD patients was lower than that of happy or sad faces, and the response to neutral faces was particularly slow. After the symptoms were relieved, the processing obstacle of neutral face was still obvious. These results indicated that, compared with healthy controls, people at risk for depression do not seem to see neutral faces as a clear sign of emotional neutrality^11^. These above studies, however, did not reflect a face perceptual deficit per se. Therefore, it is still a controversial issue whether there is a face perception disorder independent of face recognition and memory in depression.

At present, it is generally believed that face identification mainly depends on global/congfigural processing. Generally, the congfigural processes of faces include at least three aspects: (1) detecting the first-order relationship of faces (that is, two eyes above the nose and mouth); (2) processing the overall relationship (that is, the features stick together to form a Gestalt); (3) processing the second-order relationship (that is, the distance between facial features)^14^. A large amount of evidence shows that the inverted face is more difficult to recognize than the upright face, which is called "face inversion effect". This effect is mainly due to the interference of inversion on the global/holistic face processing^14^. Previous studies have found that depression patients tend to pay attention to details and individual situations rather than the overall situation, which is related to depressive symptoms, and the way of information processing may affect the development and maintenance of negative emotions of individuals with depression. Recently, using non-face stimuli Fockert and Cooper^15^ explored the correlation between visual processing and depression in the Navon task (a standard task investigating local and global visual processing), in which the global stimulus consists of many parts, and the participants are asked to report the global or local target feature shapes. It was found that participants with a low self-reported level of depression showed global processing bias (significantly faster response to the global *vs*. the local level), whereas participants with high levels of depression did not show such bias, supporting the view that depression is associated with a reduction in the tendency to give priority to global processing^15^.

The N170 component with an occipito-temporal scalp distribution is a negative deflection component, which occurs between 140 and 180 ms post stimuli onset. It is the earliest component related to facial perceptual processing, and its amplitude is reliably larger than that of other non-face stimuli (N170 face effect), and also greater than that of upright face stimuli (N170 inversion effect)^16,17^. Recently, Chen et al.^18^ adopted the visual oddball experimental paradigm and asked subjects to make selective responses to emotional facial expressions with small probability. The results showed that compared with the control group, in addition to recognizing emotional faces, the N170 amplitude of MDD patients in recognizing neutral faces was also significantly reduced and the latency was delayed^18^. Moreover, using the emotional Go/NoGo task (response to the emotional face and the opposite reaction to the neutral face), Grunewald et al.^19^ found that the N170 of MDD patients was significantly earlier than that of the control group, and the N170 elicited by neutral faces did not show the dominance effect of the right temporal occipital region in the control group^19^. To date, Yin and colleague first directly investigated the global/configural processing of faces in MDD patients using N170 as the index, showing the N170 face effect significantly larger in MDD patients than that in controls and similar the N170 inversion effect between MDD and controls^20^. However, in that study, the age range of participants were between 20 and 45 years old, that is, with some middle-aged participants. Based on the fact that face perception is modulated by the interaction between faces’ age and participants’ age (i.e., own-age bias)^21^ and that the positive attentional bias in ‘middle-aged’ MDD patients (> 30 y) was significantly lower than in ‘young’ patients (18–30 y)^22^, hence, whether there was a real dysfunction of face perception in MDD patients is till opened.

In the present study, we would investigate directly the perceptual mechanism of face processing in young MDD patients with young faces, as reflected by N170 face effect related to processing of face-related information and N170 inversion effect related to processing of face-configural information, using a passive viewing paradigm which has been widely used to investigate face perception^16,17,23^. If there is dysfunction of perceptual processing faces versus objects in MDD patients, the altered N170 face effect and/or inversion effect would be expected.

## Results

**Figure 1:**
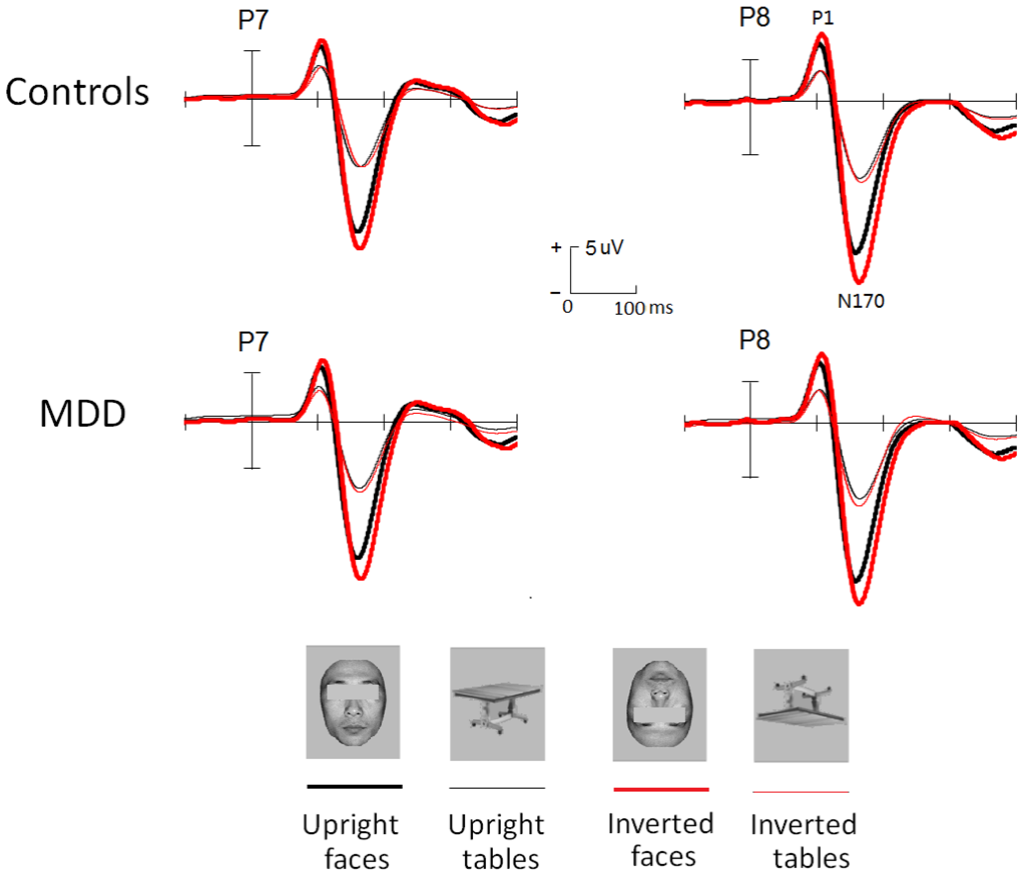
The grand ERP waveforms elicited by faces and tables in controls and MDD patients, respectively.

**Figure 2:**
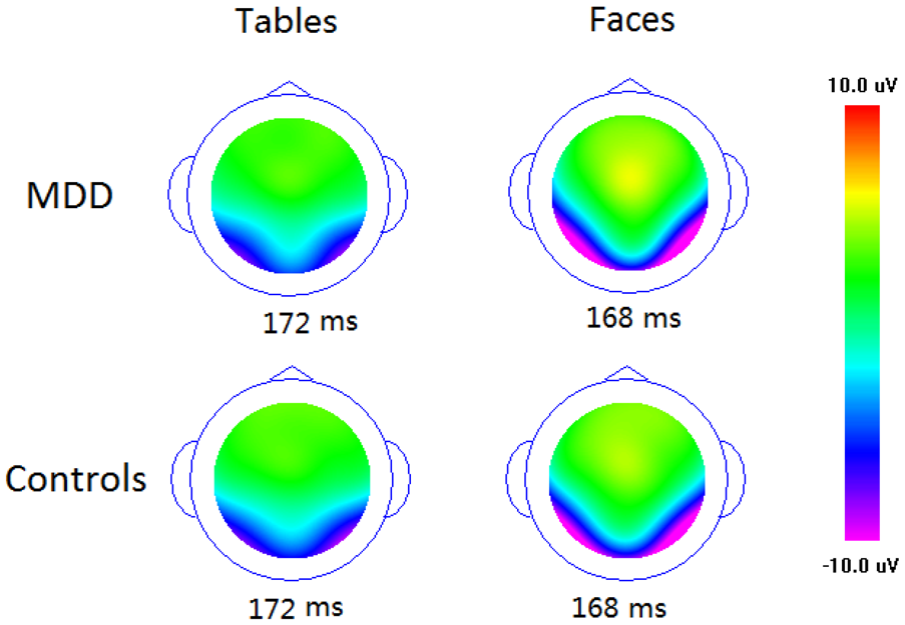
Voltage topographies at the peak of the N170 for upright faces and tables in MDD and controls, respectively.

The ERPs related to faces and objects as well as its voltage mapping was shown in Figs. 1 and 2 for two groups, respectively.

### P1 component

Across all sites and conditions, the P1 did not differ between two groups (4.5 uV and 3.9 uV for MDD and control groups, respectively, F(1,60) = 1.15, p = 0.223, partial η^2^ = 0.025; 107 ms and 106 ms for MDD and control groups, respectively, F(1,60) < 1). The analysis of the amplitude yielded several significant effects of factors with repeated measures, none of which, however, interacted with Group. The P1 elicited by faces (5.0 uV) was bigger than that elicited by tables (3.4 uV; F(1,60) = 235.3, p < 0.001, partial η^2^ = 0.797), inverted stimuli elicited a higher P1 (4.4 uV) than upright stimuli (4.0 uV; F(1,60) = 32.43, p < 0.001, partial η^2^ = 0.351) and, at the right posterior site (4.9 uV), the P1 was larger than at the left site (3.5 uV; F(1,60) = 30.585, p < 0.001, partial η^2^ = 0.338). The two-way interaction of Stimulus and Orientation was significant, F(1,60) = 11.52, p = 0.001, partial η^2^ = 0.161, and further analysis revealed that inversion effect was evident for faces (4.6 uV and 5.4 uV for upright and inverted conditions; p < 0.001) not for tables (3.3 uV and 3.5 uV for upright and inverted conditions; p = 0.296). Only two factors influenced the P1 latency significantly, and none of those interacted with Group. Its latency was longer for tables (108 ms) than faces (104 ms; F(1,60) = 35.4, p < 0.001, partial η^2^ = 0.371) and for inverted stimuli (107 ms) than for upright stimuli (104 ms; F(1,60) = 16.4, p < 0.001, partial η^2^ = 0.214).

### N170 component

The analysis of the peak latency revealed that, overall, the N170 latency was similar in the control (Mean ± SE: 169 ± 1.9 ms) and MDD (170 ± 2.0 ms; F < 1) groups. Moreover, although both the main effects of Stimulus and Orientation with repeated measures were small but significantly affected the N170 latency, none of these effects were directly modulated by Group. The latency of the N170 was longer for inverted (172 ± 1.8 ms) than for upright stimuli (164 ± 1.9 ms; F(1,60) = 62.7, p < 0.001, partial η^2^ = 0.477), and longer for tables (172 ± 2.0 ms) than for faces (168 ± 1.9 ms; F(1,60) = 12.8, p < 0.001, partial η^2^ = 0.253). No other effects reached significant level (ps > 0.1).

Similar ANOVA analysis to the N170 latency, overall, the N170 amplitude was similar in the control (Mean ± SE: − 9.3 ± 0.48 uV) and MDD (− 9.6 ± 0.48 uV; F < 1) groups. Although all three main effects of the N170 peak amplitude were significant, none of these effects were directly modulated by Group. The N170 was enhanced for faces (− 12.3 ± 0.4 uV) than tables (− 6.6 ± 0.3 uV; F(1,60) = 272.4, p < 0.001, partial η^2^ = 0.677), for inverted (− 9.9 ± 0.34 uV) than for upright (− 8.9 ± 0.33 uV; F(1,60) = 58.5, p < 0.001, partial η^2^ = 0.606) conditions and at P8 (− 10.4 ± 0.38 uV) than at P7 (− 8.5 ± 0.36 uV; F(1,60) = 11.9, p < 0.001, partial η^2^ = 0.240) sites. The two-way interaction of Stimulus * Orientation was also significant, F(1,60) = 122.5, p < 0.001, partial η^2^ = 0.763, showing evident N170 inversion effect for faces (− 2.1 ± 0.37 uV, p < 0.001) not for tables (− 0.3 ± 0.28 uV, p > 0.1). No other effects reached significant level (ps > 0.1).

## Discussion

The goal of the present study was to explore whether young MDD patients have the dysfunction of face perceptual computation in early stages of processing faces. To achieve this goal, the face perception as well as configural computation was addressed in the present study by analyzing the early ERP components related to faces and objects with upright and inverted presentations.

Across all conditions, both the P1 and the N170 components were similar in MDD patients versus in the controls. The P1 component is an early index of endogenous processing of visual stimuli, which was modulated primarily by low-level physical characteristics of the stimuli and generated in the striate and extrastriate cortex^24,25^. Although the present face effect of P1 is not the first report and could be due to insufficiently controlled low-level factors among stimulus categories, e.g., the area subtended by tables was much smaller than the area occupied by faces^23^, whether P1 is or is not face-sensitive biomarker awaits additional research. Importantly, however, the modulation of P1 by the experimental manipulations in this study was not influenced by depression.

The present study focused primarily on the N170 component reflecting the early perceptual mechanism of face processing. Replicating previous studies, we found that across groups, the N170 was higher and peaked faster in response to faces than tables, i.e., N170 face effect^16,17^. However, the N170 face effect was not impaired by depressive condition. Although the N170 elicited by facial emotion was smaller N170 in MDD^7^ and there was also evidence that depressed patients recognized neutral faces less accurately and more slowly^11^ and that the N170 face effect significantly larger in MDD patients (20–45 y) than that in controls^20^, the present study is the first direct investigation for the perceptual mechanism of face processing vs. non-face objects in young MDD patients (20–25 y), implicating that in the early perceptual stage of face perception, that is, the face is detected in the field of vision and submitted to further facial feature analysis, young MDD patients exhibit normal and complete style like healthy controls. Actually, several previous investigations did not find amplitude or latency differences for the N170 to expressions between MDD patients and normal controls^8–10^. For example, using the faces recognition task, early expression processing did not differ fundamentally reflected by the face-sensitive N170 in MDD patients versus healthy controls^10^. It should be noteworthy that the above studies mainly investigated the processing of emotional faces but the present study the face perception of neutral faces versus non-face objects, that is, the face effect isolating the facial expressions. Therefore, the early stage of face perception (including basic level and subordinate level) could be intact, which also needs further investigation (such as processing faces’ identity, gender, race, and age information) in the future.

In addition to N170 face effect, inverted faces elicited higher and delayed N170 than did upright faces^16^ and interestingly, this N170 inversion effect did not differ between MDD and healthy controls. It is widely accepted that face inversion disrupts configural processing in faces, i.e., the computation of the relationship among internal face components and their spatial location relative to face contour^26–28^. Moreover, the pattern of inversion effects in the young healthy group as well as previous N170 and behavioral studies indicate that this effect is peculiar to faces^29,30^. In line with previous study that MDD patients (20–45 y) showed the N170 inversion effect similar to controls^20^, the identical N170 inversion effect suggests that MDD patients are not affected by manipulations that interfere with configural processing of faces, at least at the early stage of face perception, regardless of patients’ age.

It should be noted that, in this study, the depression patients were all young people aged 20–25 years old and the face stimuli were also the faces of young people with similar age. Whether there is a complete face early perception processing in the middle-aged and elderly depressive patient needs further research.

Before we conclude, we should reiterate two possible aspects that limit the accounting for the present results. Firstly, the sample size is small and hence, future research should further expand the sample size, especially the applicability of the gender and/or age effects in MDD patients. Second, in this study we used unfamiliar faces to the participants. Actually, it is necessary to use unfamiliar faces to separate the initial stages of face classification, reducing the hypothetical influence of face personalization and recognition, which may be affected by memory factors. Therefore, whether the facial familiarity can modulate the perceptual processing in MDD patients awaits further investigation.

In sum, the non-impairment of face-sensitive N170 implicates intact early perceptual mechanism of processing faces in young MDD patients, relying primarily on global/configural information versus non-face objects.

## Methods

All methods were carried out in accordance with relevant guidelines and regulations. All experimental protocols were approved by the Institutional Review Board of the 960 Hospital of PLA.

### Participants

30 MDD patients (16 females; 20–25 y, mean 23.3 ± 1.2 y) and 32 age-matched healthy control participants (18 females; 21–26 y, mean 23.4 ± 1.6 y; p > 0.1) participated in this study. We recruited all MDD patients from the 960 Hospital of PLA (Jinan, China). Each patient was diagnosed with MDD, single episode, by the consensus of at least two experienced senior psychiatrists, based on the Chinese version of the Structured Clinical Interview for DSM-IV (the Diagnostic and Statistical Manual of Mental Disorders, 4th edition, Patient Edition)^31^ (Note: in the present study, we used G*Power Win 3.1 to sample size calculation with effect size d of 0.4, α err prob of 0.05 and power (1-β err prob) of 0.8^32^).

We assessed the severity of depression of patients by the HRSD-17 (17-item Hamilton Rating Scale of Depression) scores (21.8 and 2.6 for MDD and controls, respectively; p < 0.0001). The 14-item HAMA (Hamilton Anxiety Rating Scale) scores indicated the severity of comorbid anxiety (20.9 and 2.3 for MDD and controls, respectively; p < 0.0001). None of the MDD patients took antidepressants, mood stabilizers, antipsychotics, anxiolytics or hypnotics during the time of the present study.

The age-matched healthy controls had no history of any major physical or psychiatric disorders, and did not take any medication to affect the central nervous system. The exclusion criteria of two groups also included abnormal MRI results, visual or hearing impairment, neurological diseases, and drug use or addiction. This study was approved by the Institutional Review Board of the 960 Hospital of PLA, and all participants finished written informed consents before participating in this study.

### Stimuli and procedure

We followed previous methods investigating the early stage of face perception^16,19,23^. The stimuli included unfamiliar young faces with no hair, glasses, or other accessories (36 females and 36 males), tables (72), and butterflies (45), all of which were 10.58 cm * 12.70 cm gray scale photographs (Fig. 1). Five stimulus conditions were formed: upright faces and tables, inverted faces and tables, and butterflies (as targets). Using Adobe Photoshop (http://www.adobe.com), mean luminance and root-mean-square (RMS) contrast were equated across images and categories (not including the gray background in calculation). The background of all of the stimuli was a uniform gray equated to the mean stimuli luminance^23,33^. We presented all stimuli in the center of the screen, with 1.0 m away from the participants and the viewing angle of 5.05° * 6.06°.

In a dimly lit room, participants sat in a comfortable chair and were asked to keep a mental count of butterflies that occurred occasionally on the screen, while ignoring all other stimuli. This passive detection paradigm has been widely adopted in previous studies of face perceptual processing with the index of N170, which can effectively assign similar task associations to facial and non-facial stimuli^20,23^. The 333 stimuli were divided into three blocks. Each block consisted of 48 faces (24 in upright condition and 24 in inverted condition), 48 tables (24 in upright condition and 24 in inverted condition) and 14, 15 or 16 butterflies (targets), with randomized stimulus trial order. The stimulus presentation time was 300 ms, and the inter-trial interval (ITI) was 1000 ms. There was a one minute rest between two blocks.

### EEG recording

EEG signals were recorded continuously using a Neurolab digital amplifiers system (http://www.neurolab.com.cn), with a sample rate of 1000 Hz and a band pass filter of 0.1–100 Hz as well as with 30 electrodes placed according to the 10/20 system. EOG was also recorded via electrodes placed on the bilateral external canthi and the left infraorbital and supraorbital areas to monitor for eye movements and blinks. The reference electrode was the tip of the nose during recording, and a common average reference was calculated off-line.

EEGLab software (https://sccn.ucsd.edu/eeglab/index.php) was used to analyze EEG data. After EOG artifact correction using ICA method, according to the stimulus onset time, EEG signals were segmented into the epoch of 1200 ms, including 200 ms pre-stimulus onset as the baseline correction and 1000 ms post-stimulus onset. Epochs with peak-to-peak deflection exceeding ± 100 µV were excluded from averaging. After this procedure, artifact-free EEG segments were averaged according to trial conditions, respectively, and for each participant, 50 trials at least were included for each non-target condition (upright faces, inverted faces, upright tables and inverted tables) and 30 trials for butterflies. The averaged ERP waveforms were low-pass filtered at 30 Hz (24 dB/octave) (note: Because we focused on early ERP components and the stimulus presentation lasted 300 ms, if segments were rejected for artifacts in the ITI following face presentation, this could unnecessarily reduce trial counts by rejecting usable trials with clean data during face presentation. Therefore, we also processed data with the epoch of 500 ms, including 100 ms pre-stimulus onset as the baseline correction and 400 ms post-stimulus onset and found the similar results.)

### Data analysis

According to previous studies^16,17,20,23^, the N170 component was assessed at left (P7) and right occipital-temporal sites (P8). In addition, we have also analyzed the preceding P1 component at the same locations. Based on visual inspection for the grand averaged ERP waveform as well as previous studies^16,20,23^, the peak amplitudes and latencies were measured automatically between 70 and 120 ms and between 120 and 200 ms for P1 and N170, respectively. These measures were submitted to mixed-model ANOVAs, with Group (controls, MDD) as a between-subject factor, and Stimulus (face, tables), Orientation (upright, inverted) and Site (P7, P8) as within-subject factors. We used the Greenhouse–Geisser epsilon correction factor to adjust the degrees of freedom for all ANOVAs and Bonferroni corrections for multiple comparisons. Paired sample t tests (two-tailed) were used for planned comparisons.

## Data Availability

The raw data in Prof. Lun Zhao’s Lab supporting the conclusions of this article will be made available by the authors, without undue reservation.
